# Kidney disease risk factors associate with urine biomarkers concentrations in HIV-positive persons; a cross-sectional study

**DOI:** 10.1186/s12882-018-1192-y

**Published:** 2019-01-03

**Authors:** Anthony N. Muiru, Michael G. Shlipak, Rebecca Scherzer, William R. Zhang, Simon B. Ascher, Vasantha Jotwani, Carl Grunfeld, Chirag R. Parikh, Derek Ng, Frank J. Palella, Ken Ho, Seble Kassaye, Anjali Sharma, Mardge Cohen, Ruibin Wang, Qibin Qi, Michelle M. Estrella

**Affiliations:** 1 Kidney Health Research Collaborative, Department of Medicine, San Francisco Veterans Affairs Medical Center and University of California, 533 Parnassus Avenue, U404, Box 0532, San Francisco, CA 94143 USA; 2 Department Epidemiology, and Biostatistics, University of California, San Francisco, CA USA; 3 Department of Medicine, University of California, Los Angeles, CA USA; 4 Department of Medicine, Section of Nephrology, Yale University, New Haven, CT USA; 5 Department of Epidemiology, Johns Hopkins Bloomberg School of Public Health, Baltimore, MD USA; 6 Division of Infectious Disease, Northwestern University Feinberg School of Medicine, Chicago, IL USA; 7 Division of Infectious Diseases, Department of Medicine, University of Pittsburgh, Pittsburgh, PA USA; 8 Georgetown University Medical Center, Washington, DC, USA; 9 Department of Epidemiology and Population Health, Albert Einstein College of Medicine, Bronx, NY USA; 10 Department of Medicine, Stroger Hospital and Rush University, Chicago, IL USA

**Keywords:** Urine biomarkers, Kidney injury, HIV infection, Multicenter AIDS cohort study (MACS), Women’s interagency HIV study (WIHS)

## Abstract

**Background:**

HIV-positive persons bear an excess burden of chronic kidney disease (CKD); however, conventional methods to assess kidney health are insensitive and non-specific for detecting early kidney injury. Urinary biomarkers can detect early kidney injury, and may help mitigate the risk of overt CKD.

**Methods:**

Cross-sectional study of HIV-positive persons in the Multicenter AIDS Cohort Study and the Women’s Interagency HIV Study. We measured levels of 14 biomarkers, capturing multiple dimensions of kidney injury. We then evaluated associations of known CKD risk factors with urine biomarkers using separate multivariable adjusted models for each biomarker.

**Results:**

Of the 198 participants, one third were on HAART and virally suppressed. The vast majority (95%) had preserved kidney function as assessed by serum creatinine, with a median eGFR of 103 ml/min/1.73 m^2^ (interquartile range (IQR): 88, 116). In our multivariable analyses, the associations of each CKD risk factor with urinary biomarker levels varied in magnitude. For example, HIV viral load was predominantly associated with elevations in interleukin(IL)-18, and albuminuria, while higher CD4 levels were associated with lower monocyte chemoattractant protein-1 (MCP-1) and β2-microglobulin. In contrast, older age was significantly associated with elevations in α1-microglobulin, kidney injury marker-1, clusterin, MCP-1, and chitinase-3-like protein-1 levels, as well as lower epidermal growth factor, and uromodulin levels.

**Conclusions:**

Among HIV-positive persons, CKD risk factors are associated with unique and heterogeneous patterns of changes in urine biomarkers levels. Additional work is needed to develop parsimonious algorithms that integrate multiple biomarkers and clinical data to discern the risk of overt CKD and its progression.

**Electronic supplementary material:**

The online version of this article (10.1186/s12882-018-1192-y) contains supplementary material, which is available to authorized users.

## Background

The improved life expectancy among treated HIV-positive patients has been tempered by the excess burden of age-related non-infectious co-morbidities, including chronic kidney disease (CKD) [1–3]. In this population, CKD results not only from traditional risk factors, such as diabetes and hypertension, but also from human immunodeficiency virus (HIV)-related risk factors [4, 5], including uncontrolled viremia [6], chronic co-infection with hepatitis C virus (HCV) [7], and exposure to potentially nephrotoxic antiretroviral (ART) medications [8, 9]. These risk factors culminate in excess risk of CKD among HIV-positive persons compared to the general population [3, 5]. Importantly, CKD significantly contributes to excess morbidity and mortality experienced by HIV-positive individuals [10–12]. Earlier detection of kidney damage could potentially help mitigate the risk of overt CKD and its consequences. Unfortunately, conventional indicators of kidney disease, including serum creatinine and proteinuria, are relatively insensitive and non-specific for detecting early kidney injury [11, 13]. These indicators of kidney disease become abnormal only when significant damage or dysfunction has occurred, and they do not localize the specific site of injury within the nephron [14, 15].

In contrast, novel urinary biomarkers are emerging as valid markers of early kidney injury [16]. These biomarkers have been demonstrated to predict longitudinal kidney function as well as other adverse outcomes in specific clinical scenarios, such as following major cardiac surgery [17], among kidney transplant recipients [18, 19], and among HIV-positive and negative ambulatory populations [20–26]. However, CKD pathogenesis often involves multiple risk factors that may cause injury at diverse parts of the nephron and contribute to progressive loss of kidney function. Therefore, a set of complementary urinary biomarkers, rather than a single biomarker, is likely needed to capture these multiple dimensions of kidney injury and to distinguish the site-specific risk factors within the nephron. Ideally, levels of these biomarkers would also prognosticate CKD risk, and thus inform clinical decision-making in a variety of clinical settings encountered in the care of HIV-positive persons [16, 27].

To evaluate whether each CKD risk factor has a distinct pattern of kidney injury, we examined their associations with a panel of urine biomarkers of kidney injury among HIV-positive individuals who were not on tenofovir disoproxil fumarate (TDF) in the Multicenter AIDS Cohort Study (MACS) and the Women’s Interagency HIV Study (WIHS). We hypothesized that each CKD risk factor would be associated with levels of a unique set of urinary biomarkers, indicating a distinct profile of kidney injury and dysfunction.

## Methods

### Study population and study design

The MACS and WIHS are ongoing, longitudinal prospective observational studies of men and women, respectively, who are either infected with HIV or considered at high-risk for acquiring HIV. The MACS and WIHS cohorts share similar research goals, which include characterizing the long-term benefits and adverse effects of ART. Both cohorts have been previously detailed elsewhere [28–30]. Briefly, the MACS enrolled 7355 men who have sex with men between 1984 and 2017 from four study sites: Baltimore, MD/ Washington, D.C.; Chicago, IL; Los Angeles, CA; and Pittsburgh, PA/Columbus, Ohio. The WIHS initially enrolled a total of 4909 women in 1994–1995 and 2001–2002 from six study sites: Bronx and Brooklyn, NY; Chicago, IL; Los Angeles and San Francisco, CA; and Washington, D.C. The WIHS subsequently enrolled an additional 1216 women between 2011 and 2015 from the initial set of sites, with the addition of participants from Atlanta, GA, Birmingham, AL, Jackson, MS, Chapel Hill, NC, and Miami, FL. In both cohorts, standardized questionnaires to obtain sociodemographic and clinical information are administered during semi-annual study visits. In addition, physical examinations and collection of biological specimens are performed during these visits. At certain visits, urine specimens were also collected and stored in each cohort. The current cross-sectional study utilized data from an observational study evaluating the association of TDF-based ART with changes in urinary biomarkers levels. Because we were interested in the effect of traditional CKD risk factors on urinary biomarkers, we evaluated participants just prior to initiation of TDF—a known nephrotoxin [31].

### Measurement of urine biomarkers of kidney injury

Clean catch urine specimens were collected prospectively, refrigerated immediately after collection, and subsequently centrifuged. Supernatants were then stored in 1-mL aliquots at − 80 °C until biomarker measurement was undertaken, without prior freeze-thaw. We measured levels of 14 urine biomarkers, each hypothesized to indicate a distinct dimension of kidney injury and dysfunction. Although the precise pathogenic mechanisms of these biomarkers are incompletely understood, we conceptualized them as follows based on prior studies: 1) glomerular/ endothelial injury: albumin-to-creatinine ratio (ACR) and osteopontin (OPN); 2) proximal tubular dysfunction: cystatin C (CysC), α1-microglobulin (α1m), and β2-microglobulin (β2m); 3) tubular injury: kidney injury molecule-1 (KIM-1), trefoil factor 3 (TFF3); clusterin, neutrophil gelatinase-associated lipocalin (NGAL) and interleukin (IL)-18; 4) loop of Henle dysfunction: uromodulin (UMOD); and 5) tubulointerstitial injury and fibrosis: monocyte chemoattractant protein-1 (MCP-1), epidermal growth factor (EGF), and [32–34]. All urine biomarkers were measured using multiplex immunoassays from Meso Scale Discovery (MSD, Rockville, MD), except urine creatinine which was measured using the Roche enzymatic creatinine assay (Roche Diagnostics, Indianapolis, IN) and α1m, which was measured using a commercial assay (Siemens BN II Nephelometer, Munich, Germany). Intra-assay coefficients of variation were < 15% for all biomarkers (Additional file 1: Table S1).

### Definitions of risk factors for CKD

We evaluated the following CKD risk factors: 1) age, 2) self-reported race/ethnicity, 3) self-reported cigarette use, 4) diabetes mellitus, 5) hypertension, 6) HCV co-infection, 7) plasma HIV-1 RNA (viral load), and 8) CD4+ count. Consistent with national guideline definitions and with prior MACS and WIHS analyses, diabetes mellitus was defined as: hemoglobin A1c ≥6.5%, fasting plasma glucose ≥126 mg/dL (7 mmol/L) or self-reported history of diabetes with self-reported use of anti-diabetic medications [35]. Hypertension was defined as: two consecutive measurements of systolic blood pressure (SBP) ≥140 mmHg, or diastolic blood pressure (DBP) ≥90 mmHg, or self-reported history of hypertension with self-reported use of an antihypertensive medication [36]. HCV infection was determined by detectable HCV RNA following a positive HCV antibody result. Detectable HIV viral load was defined as plasma HIV-1 RNA ≥ 80 copies/mL. In the MACS, plasma HIV RNA concentrations were measured using the Roche COBAS Ultrasensitive Amplicor HIV-1 monitor assay (lower level of detection (LLD) of 50 copies /mL), or the Roche Taqman HIV-1 Test (LLD of 20 copies/mL). In the WIHS, plasma HIV RNA was measured using the Roche COBAS AmpliPrep/COBAS TaqMan HIV-1 Test (LLD of 20 or 48 copies HIV RNA/mL). Serum creatinine-based estimated glomerular filtration rate (eGFR) was calculated using the CKD-EPI equation [37].

### Statistical analysis

Demographic and clinical characteristics were summarized overall, and stratified by cohort. We evaluated associations of risk factors with biomarker levels in a series of models: 1) separate unadjusted linear regression models; 2) multivariable simultaneous linear equations; and 3) multivariable sparse group least absolute shrinkage and selection operator (MSG-LASSO). In all models, biomarker concentrations were log-transformed to normalize their distributions, and results were back-transformed to produce estimated percentage differences in biomarker levels attributable to each risk factor. We controlled for urine creatinine in all analyses to account for urine tonicity. Additional co-variates included other race, Hispanic race, past smoking, and history of ART use.

We used separate linear regression models for each biomarker to evaluate unadjusted risk factor associations with robust Huber-Weight standard errors. We then used multivariable simultaneous linear equations (constructed with three-stage least squares) to account for correlations between urine biomarkers. This method is more appropriate than individual regression models given the relatedness of the biomarker measurements. In a final step, rather than using traditional multiple comparison adjustments to control the type I error rate, we modeled biomarkers in combination using MSG-LASSO method for variable selection [38]. To obtain corresponding 95% confidence intervals and *p*-values for the LASSO-selected variables, we then modeled biomarkers in combination using multivariable linear regression analysis with an L1 penalty.

The LASSO analysis was implemented using the R package *MSGLasso*. All other analyses were performed using the SAS system, version 9.4 (SAS Institute, Inc., Cary, NC).

## Results

Of 198 HIV-positive participants, the majority (64%) were black, over half (56%) were women, and the median age was 48 years (interquartile range [IQR]: 41, 54) (Table 1). Median CD4+ count was 483 cells/mm^3^ ([IQR]: 338, 682), 29% of persons had undetectable HIV viral load (HIV RNA <  80 copies/mL), 33% were on ART, 48% were hypertensive, 17% had diabetes, and 17% were co-infected with HCV. Majority (95%) of the participants had preserved kidney function as assessed by serum creatinine with a median eGFR of 103 ml/min/1.73 m^2^ (IQR: 88, 116). In addition, participants had minimal albuminuria, with only 8% having an ACR > 30 mg/g. Characteristics within each cohort are presented in Table 1.

**Table 1:** Sociodemographic and clinical characteristics of HIV-positive individuals, by cohort

Parameter	Overall (*n* = 198)	WIHS (*n* = 111)	MACS (*n* = 87)
Age,y	48 (41, 54)	46 (40, 53)	49 (44, 56)
Race/ethnicity			
Black	126 (64)	87 (78)	39 (45)
White	59 (30)	15 (14)	44 (50)
Other	13 (6)	9 (8)	4 (5)
Hispanic	29 (15)	20 (18)	9 (10)
Smoking			
Current	73 (37)	46 (41)	27 (31)
Past	62 (31)	25 (23)	37 (43)
Never	62 (31)	40 (36)	22 (26)
Diabetes mellitus	32 (17)	20 (18)	12 (17)
Systolic BP, mmHg	126 (114, 137)	121 (110, 134)	129 (117, 137)
Diastolic BP, mmHg	77 (71, 86)	74 (69, 85)	80 (72, 87)
Hypertension	93 (48)	56 (50)	37 (44)
Antihypertensive use	70 (35)	45 (41)	25 (29)
Statin use	31 (16)	17 (15)	14 (17)
History of CVD	13 (7)	4 (4)	9 (10)
BMI, kg/m^2^	27 (23, 32)	29 (24, 34)	25 (23, 28)
Waist Circumference, cm	94 (83, 104)	96 (82, 106)	92 (84, 102)
Current HAART	66 (33)	31 (28)	35 (40)
Current NRTI	67 (34)	31 (28)	36 (41)
Current NNRTI	34 (17)	13 (12)	21 (24)
Current PI	30 (15)	14 (13)	16 (18)
Current CD4+ count, cells/mm^3^	483 (338, 682)	485 (314, 667)	465 (387, 716)
History of AIDS	26 (13)	23 (21)	3 (3)
Current HIV RNA, < 80 copies/mL	56 (29)	24 (22)	32 (37)
Hepatitis C virus seropositive	33 (17)	20 (18)	13 (15)
Estimated GFR	103 (88, 116)	103 (85, 117)	104 (92, 116)
ACR,mg/g	3.2 (1.9, 7.1)	3.5 (2.0, 12.0)	3.0 (1.8, 5.7)

Data are presented as Median (IQR) or numbers (percent). *WIHS* Women’s Interagency HIV Study, *MACS* Multicenter AIDS Cohort Study, *BP* Blood pressure, *CVD* Cardiovascular disease, *BMI* Body Mass Index, *HAART* Highly active antiretroviral therapy, *NRTI* Nucleoside Reverse Transcriptase Inhibitors, *NNRTI* Non-Nucleoside Reverse Transcriptase Inhibitors, *PI* Protease inhibitors, *GFR* Glomerular filtration rate, *ACR* albumin to creatinine ratio

As displayed in Table 2, we observed distinct patterns of risk factors associated with each biomarker in unadjusted analyses. For example, black race, current smoking, diabetes, HCV-seropositivity, and higher HIV viral load were individually associated with higher levels of IL-18, whereas higher CD4+ count was associated with lower IL-18 levels. Conversely, when evaluated from the perspective of each CKD risk factor, the associated biomarkers had heterogeneous patterns and were varied in magnitude. For example, current smoking had the strongest association with elevations in α1m, and the magnitude of the point estimate was 3-fold the elevation observed per 10-year increase in age (106% greater a1m for current smoking versus 36% for age).

**Table 2:** Unadjusted associations of traditional and HIV-related risk factors with urine biomarker levels among HIV-positive participants

	α1m	β2-m	IL-18	KIM-1	TFF3	Clusterin	NGAL	MCP-1	EGF	UMOD	ACR	CysC	OPN	YKL-40
Risk Factors	% Estimate (95% CI)	% Estimate (95% CI)	% Estimate (95% CI)	% Estimate (95% CI)	% Estimate (95% CI)	% Estimate (95% CI)	% Estimate (95% CI)	% Estimate (95% CI)	% Estimate (95% CI)	% Estimate (95% CI)	% Estimate (95% CI)	% Estimate (95% CI)	% Estimate (95% CI)	% Estimate (95% CI)
Age (per decade)	36 (21, 52)	11 (−10, 37)	10 (−1, 22)	42 (27, 58)	9 (−15, 39)	30 (16, 47)	21 (4, 40)	26 (16, 35)	−14 (−19, −7)	−21 (−30, − 11)	23 (6, 42)	3 (−4, 11)	5 (−4, 15)	18 (2, 37)
Black Race	16 (−10, 49)	131 (39, 284)	31 (3, 68)	− 25 (−44, 0.7)	170 (60, 358)	− 7 (− 33, 28)	102 (44, 181)	9 (− 12, 36)	− 25 (− 37, − 10.8)	−8 (− 34, 29)	37 (−0.6, 90)	3 (− 14, 23)	−9 (− 25, 10)	61 (15, 124)
Current smoking	106 (61, 164)	57 (− 4, 156)	38 (9, 75)	5 (− 19, 35)	59 (− 7, 17)	50 (11, 102)	29 (− 11, 86)	8 (−13, 35)	−4 (− 19, 14)	−2 (− 26, 30)	39 (− 4, 101)	18 (0.13, 39)	−12 (− 28, 9)	4 (−26, 47)
Diabetes	29 (− 1, 68)	−54 (− 76, − 10)	38 (8, 76)	27 (− 2, 65)	−28 (−64, 44)	28 (−6, 75)	52 (1.6, 126)	16 (− 8, 47)	−20 (− 34, − 3)	− 33 (− 49, − 11)	39 (− 9, 114)	− 14 (− 31, 8)	6 (− 15, 32)	3 (− 34, 61)
Hypertension	20 (−2, 47)	5 (−31, 59)	6 (− 14, 30)	14 (− 8, 42)	16 (− 26, 83)	11 (− 13, 43)	4 (−23, 41)	3 (− 14, 23)	−24 (− 34, − 13)	4 (− 17, 31)	56 (16, 111)	−2 (− 14, 11)	− 4 (− 19, 14)	21 (− 11, 65)
HCV-seropositive	62 (25, 111)	107 (25, 245)	56 (17, 107)	29 (−4.0, 72)	139 (27, 351)	33 (−4.0, 84)	34 (− 6, 90)	3 (− 21, 35)	−21 (− 36, − 49)	−8 (− 30, 22)	−28 (− 64, 44)	12.5 (− 7, 36)	−4 (− 299, 29)	39 (− 1.6, 97)
HIV RNA (per 10-fold higher)	4 (− 4, 11)	26 (9, 45)	18 (10, 27)	4 (−5, 13)	6 (−10, 23)	− 4 (− 13, 6)	0.7 (− 9, 12)	3 (− 4, 9)	− 0.7 (− 5, 49)	6 (− 2, 15)	7 (−3, 19)	6 (2, 11)	− 0.8 (− 7, 6)	9 (− 2, 21)
CD4+ count (per (doubling)	−12 (− 22, − 1)	−35 (− 49, − 18)	− 21 (− 30, − 11)	−7 (− 17, 4)	−3 (− 26, 29)	−0.4 (− 15, 16)	2 (−16, 24)	− 15 (− 24, − 5)	2 (− 5, 10)	−2 (− 14, 12)	−11.0 (− 24, 4)	− 9 (−17, −1)	0.9 (− 9, 12)	−15 (− 30, 1.4)

Estimates from linear regression models adjusted for urine creatinine. *α1m* α1-microglobulin, *β2m* β2-microglobulin, *IL-18* interleukin 18, *KIM-1* kidney injury marker-1, *TFF3* trefoil factor 3, *NGAL* neutrophil gelatinase-associated lipocalin, *MCP-1* monocyte chemoattractant protein-1, *EGF* epidermal growth factor, *UMOD* uromodulin, *ACR* albumin-to-creatinine ratio, *CysC* cystatin C, *OPN* osteopontin, *YKL-40* chitinase-3-like protein-1

Among CKD risk factors, older age showed statistically significant associations with nearly all dimensions of kidney injury. In unadjusted analyses, older age was significantly associated with: 1) higher urinary marker levels of proximal tubular dysfunction (α1m); 2) higher urinary marker levels of tubular injury (KIM-1, clusterin, and NGAL); 3) lower UMOD levels, indicative of loop of Henle dysfunction; 4) greater albuminuria, indicative of glomerular injury; and 5) higher YKL-40, higher MCP-1, and 6) lower EGF concentrations, indicative of tubulointerstitial fibrosis. In contrast, HIV viral load was predominantly associated with increased levels of IL-18, β2-m and CysC. Higher CD4+ levels were associated with lower levels of α1m, β2-m, IL-18, MCP-1 and CysC. Blacks compared to non-blacks had higher NGAL, β2m, IL-18, TFF3, and YKL-40 levels, they also had lower levels of EGF in unadjusted analyses.

**Fig. 1:**
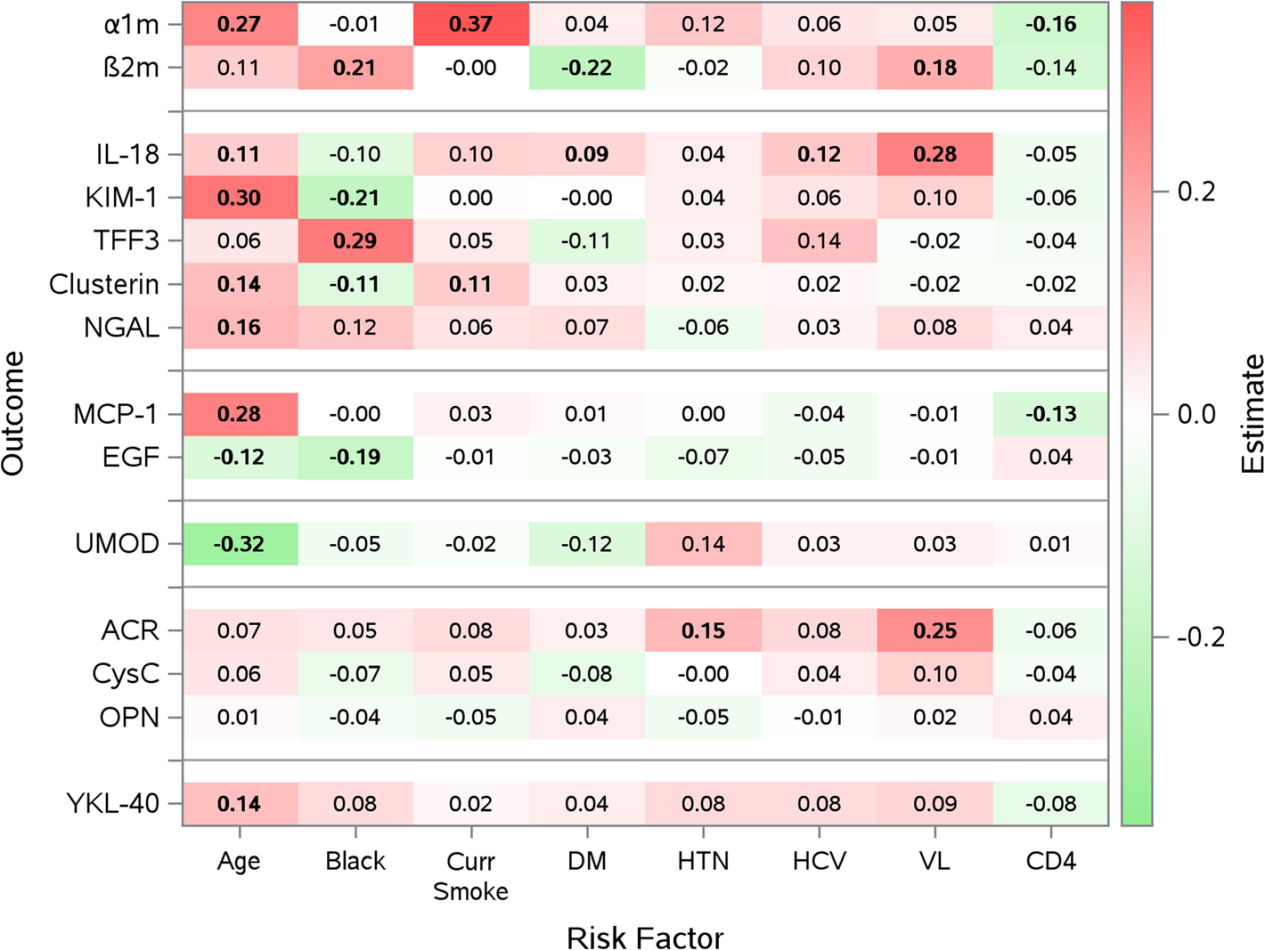
Adjusted associations of CKD risk factors with urinary biomarker concentrations by multivariable simultaneous linear equations. Models were adjusted for urine creatinine, Hispanic ethnicity, other race, past smoking, and history of ART use in addition to the CKD risk factors listed above. Statistically significant estimates are shown in bold. Red shaded cells indicate factors associated with higher urine biomarker levels, green shaded cells indicate factors associated with lower urine biomarker levels. α1m: α1-microglobulin; β2m: β2-microglobulin; IL-18: interleukin 18; KIM-1: kidney injury marker-1; TFF3: trefoil factor 3; NGAL: neutrophil gelatinase-associated lipocalin; MCP-1: monocyte chemoattractant protein-1; EGF: epidermal growth factor; UMOD: uromodulin; ACR: albumin-to-creatinine ratio; CysC: cystatin C; OPN: osteopontin; YKL-40: chitinase-3-like protein-1; Curr Smoke; current smoking DM; Diabetes, HTN; hypertension, HCV: Hepatitis C virus, VL: HIV viral load in copies/mL

**Fig. 2:**
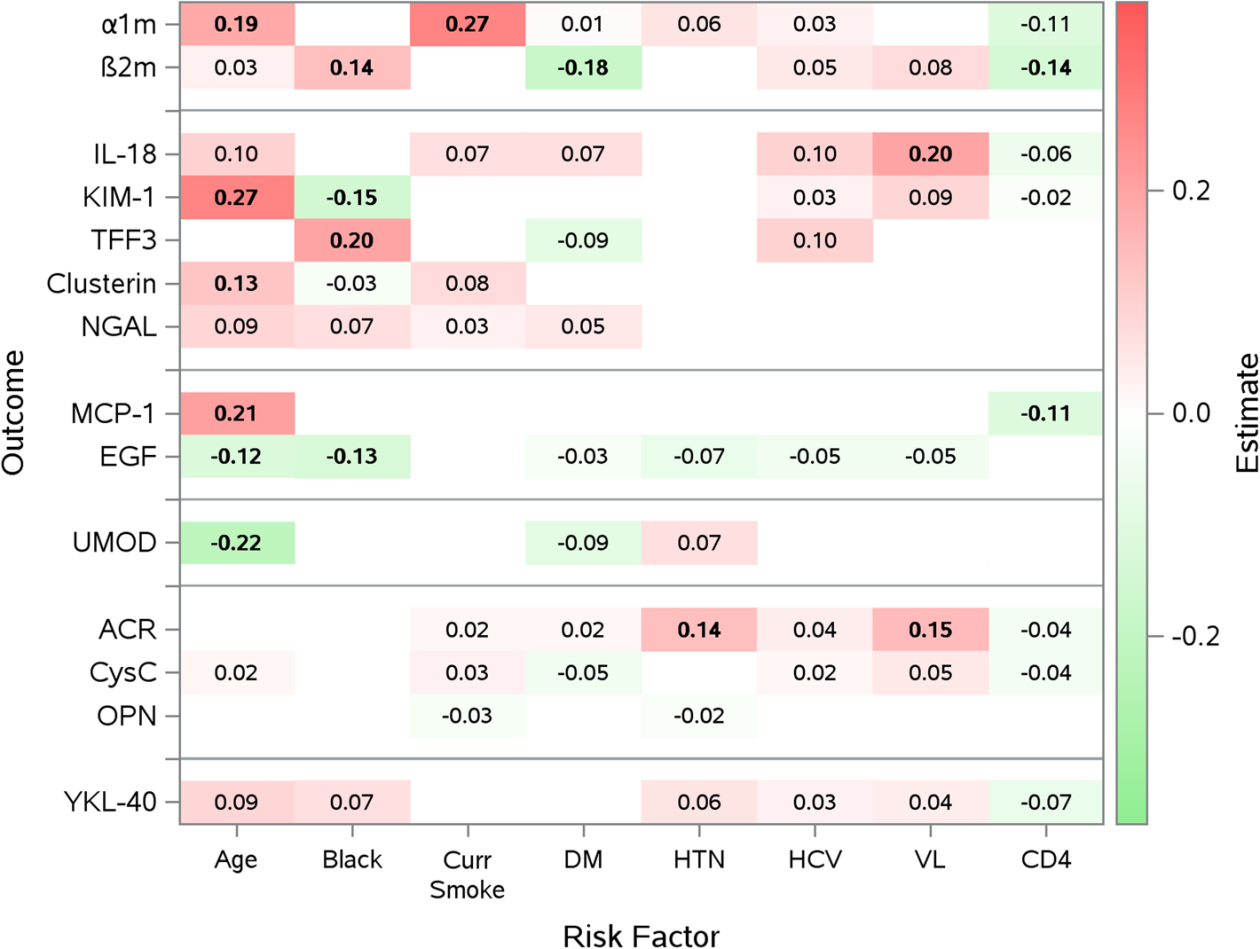
Parsimonious model by multivariable sparse group least absolute shrinkage and selection operator (MSG-LASSO) method for variable selection. Numbers within each cell represent standardized beta coefficients. These can be interpreted like correlation coefficients (scaled from − 1 to + 1). e.g., a 1 standard deviation (SD) older age is associated with 0.27 SD higher α1m. Red shaded cells indicate factors associated with higher urine biomarker levels, green shaded cells indicate factors associated with lower urine biomarker levels. The degree of shading correlates with the magnitude of the standardized beta coefficients. Statistically significant estimates are shown in bold. α1m: α1-microglobulin; β2m: β2-microglobulin; IL-18: interleukin 18; KIM-1: kidney injury marker-1; TFF3: trefoil factor 3; NGAL: neutrophil gelatinase-associated lipocalin; MCP-1: monocyte chemoattractant protein-1; EGF: epidermal growth factor; UMOD: uromodulin; ACR: albumin-to-creatinine ratio; CysC: cystatin C; OPN: osteopontin; YKL-40: chitinase-3-like protein-1; Curr Smoke; current smoking DM; Diabetes, HTN; hypertension, HCV: Hepatitis C virus, VL: HIV viral load in copies/mL

As shown in Figs 1 and 2 although attenuated, many of the risk factor and biomarker level associations persisted after multivariable adjustment in simultaneous linear equations (Fig. 1) and after MSG-LASSO selection **(**Fig. 2). For instance, older age remained significantly associated with markers of proximal tubular dysfunction and injury, loop of Henle dysfunction and tubulointerstitial fibrosis, even after controlling for all other risk factors in the model. However, while age was significantly associated with greater YKL40 levels in the multivariable model (+ 0.14, *p* = 0.04), this association weakened after LASSO selection (+ 0.09, *p* = 0.1). In addition, age was no longer significantly associated with ACR in the multivariable model (+ 0.07, *p* = 0.4) or LASSO selection (0.00, *p* = 0.9). HIV viral load remained predominantly associated with IL-18 and ACR levels, while higher CD4+ counts remained associated with lower MCP-1 levels. Of note, higher CD4+ counts were associated with lower α1m levels (− 0.16, *p* = 0.02) in the initial multivariable model but not in the final MSG-LASSO (− 0.11, *p* = 0.08), and lower β2m levels in the MSG-Lasso (− 0.14, *p* = 0.04) but not in the multivariable model (− 0.14, *p* = 0.08).

## Discussion

In this cross-sectional analysis of well-characterized HIV-positive men and women, we observed that each traditional and HIV-specific CKD risk factor was associated with levels of a unique set of complementary urinary biomarkers, which varied in magnitude. Of note, this study population had preserved kidney function as assessed by serum creatinine, yet CKD risk factors were associated with alterations in levels of urinary biomarkers, highlighting that conventional methods of assessing kidney health may not adequately capture early kidney injury [14, 15]. Most of these biomarkers have been linked to longitudinal declines in kidney function, which suggests that the biomarker panel is reflecting incipient kidney disease risk at an earlier stage than can be clinically detected with current methods [16, 21–23, 25, 27].

The pathophysiology of CKD is complex, particularly among HIV-positive persons, and involves multiple risk factors. These risk factors may simultaneously contribute to injury at various segments of the nephron, eventually leading to progressive loss of kidney function. The association between a particular CKD risk factor and a specific pattern of change in levels of urinary biomarkers can help to discriminate the contribution of each risk factor towards kidney injury in a variety of clinical settings encountered in HIV care. For instance, current cigarette smoking was predominantly associated with elevations in α1m in our final models, while HIV viral load was predominantly associated with elevation of IL-18 and ACR. Higher concentrations of urine α1m in a currently smoking, HIV-positive patient may distinguish smoking as the primary kidney insult, while elevation in IL-18 along with ACR in the same patient may suggest HIV viremia as the predominant culprit. Distinguishing the extent and nature of the contribution of each risk factor towards kidney injury can inform clinical decision-making, such as intensification of renal-protective therapy, aggressive treatment of modifiable risk factors, and identification and removal of potential nephrotoxins.

In addition, assessment of urinary biomarker levels can help localize the site of injury within the nephron. For instance, hypertension was associated with higher ACR levels. Hypertension is known to cause glomerular endothelial damage, as reflected by albuminuria [39]. Older age was associated with changes in urinary biomarkers indicative of injury spanning the entire nephron, including proximal tubule dysfunction (α1m), tubular injury (KIM-1, clusterin and NGAL), loop of Henle dysfunction (UMOD), and tubulointerstitial injury and fibrosis (YKL-40). We also noted that older age was associated with lower EGF levels, a protein considered a surrogate marker for regenerative tubular reserve that may facilitate the kidney’s ability to recover from injury and slow progression of CKD [40]. However, dysregulation of this repair pathway, reflected by high urinary EGF excretion, may promote fibrosis, inflammation and progression of CKD [41]. Our observed association between older age and this extensive panel of kidney injury markers that indicate injury across all the regions of the nephron are consistent with well-described structural and functional changes seen in the aging kidney including decreased number of functional glomeruli [42], proximal tubule shrinkage [43], tubular atrophy and interstitial fibrosis [44]. Since this cohort was middle-aged, similar studies should be conducted among HIV-negative persons to determine whether the effects of age on the kidney are accelerated by HIV infection.

We have previously reported the association of HIV viremia with increased urinary IL-18 and ACR levels [45], and we confirmed these findings in this analysis that included both men and women. Although we demonstrated consistent associations between HIV viral load and urinary biomarker levels, we did not find that black race was associated with either ACR or IL-18 levels, as previously reported [45]. Similarly, we did not observe an association between diabetes and ACR levels in our study. There are several potential explanations for this observation. First, only 32 of studied participants in current analysis were diabetic so we may have lacked sufficient power to detect differences in urinary biomarker levels between participants with and without diabetes. Second, participants in our study had diabetes for a short period of time, with a median duration of diabetes of 6.5 years (IQR 2.3–9.3). Furthermore, in WIHS the median hemoglobin A1c was 6.7 (IQR 5.9–7.8), indicating excellent glycemic control and at least 40% of diabetic patients were treated with renin angiotensin aldosterone system inhibitors. All these factors have been associated with lower ACR and improved renal outcomes in clinical trials [46].

Our results should be interpreted in the context of our study’s limitations. First, this is a cross-sectional study so causative associations between CKD risk factors and urinary biomarker levels cannot be assumed. Second, participants included in this study were individuals who were not on TDF, and our results may not be generalizable to patients on such ARTs. Third, we lacked kidney biopsy results to confirm the presence of kidney injury histologically; however, urinary biomarkers selected for inclusion in this analysis have all been shown to be associated with acute kidney injury, longitudinal kidney function decline and mortality [17, 20–25]. Finally, our sample size may have been insufficient to detect findings with moderate effect sizes, especially when using the very conservative LASSO approach.

## Conclusions

We have shown that each known CKD risk factor is associated with a distinct pattern of changes in urine biomarkers levels. While our findings highlight the potential clinical utility of routine measurement of multiple biomarker levels, our findings require validation in larger, more diverse patient populations. Evaluation of the predictive performance of biomarker measurement in the patient populations described herein address a necessary step in the ascertainment of the potential value of urinary biomarker level measurement for use in broader clinical settings [47]. Ultimately, parsimonious algorithms that integrate multiple biomarker levels results along with clinical data will be critical for translating these novel diagnostic strategies into standard clinical practice.

## Additional file

Additional file 1: **Table S1.** Urine biomarker Assay information. Table showing each biomarker assay information including intra-assay coefficients of variation for all biomarkers used in this analysis. (DOCX 16 kb)

## Abbreviations

ACR: Albumin-to-creatinine ratio
ART: Antiretroviral
CKD: Chronic kidney disease
CysC: Cystatin C
DBP: Diastolic blood pressure
EGF: Epidermal growth factor
eGFR: Estimated glomerular filtration rate
HCV: Hepatitis C virus
HIV: Human immunodeficiency virus
IQR: Interquartile range
KIM-1: Kidney injury molecule-1
LLD: Lower level of detection
MACS: Multicenter AIDS Cohort Study
MCP-1: Monocyte chemoattractant protein-1
MSD: Meso Scale Discovery
MSG-LASSO: Multivariable sparse group least absolute shrinkage and selection operator
NGAL: Neutrophil gelatinase-associated lipocalin
OPN: Osteopontin
SBP: Systolic blood pressure
TDF: Tenofovir disoproxil fumarate
TFF3: Trefoil factor 3
UMOD: Uromodulin
WIHS: Women’s Interagency HIV Study
α1m: α1-microglobulin
β2m: β2-microglobulin
