# Supplementary material for: Kidney disease risk factors associate with urine biomarkers concentrations in HIV-positive persons; a cross-sectional study
Source: BMC Nephrol. 2019 Jan 3;20:4. doi: 10.1186/s12882-018-1192-y (PMC6318986; doi:10.1186/s12882-018-1192-y)
Supplement: Supplementary file 1 — Table S1. Urine biomarker Assay information. Table showing each biomarker assay information including intra-assay coefficients of variation for all biomarkers used in this analysis. (DOCX 16 kb) [file 12882_2018_1192_MOESM1_ESM.docx]

**S1Table: Urine Biomarker Assay Information**

| **Panel (manufacturer)** | **Assays** | **Study** | **Observed Range** | **Observed Average** | **Inter-Assay CV% Average** |
| --- | --- | --- | --- | --- | --- |
| MSD Kidney Injury Panel 5 (Meso Scale Diagnostics, Gaithersburg, MD) | Albumin | WIHS | 315 - ≥ 60,240 ng/mL | 14,422 ng/mL | 10.19% |
|  |  | MACS | 310 - ≥ 60,240 ng/mL | 15,113 ng/mL | 7.15% |
|  | β2m | WIHS | 0.67 - 20,249 ng/mL | 1,070 ng/mL | 9.09% |
|  |  | MACS | 2.21 - 33,797 ng/mL | 1,337 ng/mL | 8.77% |
|  | CysC | WIHS | 4.29 - 21,210 ng/mL | 132.91 ng/mL | 7.60% |
|  |  | MACS | 2.56 - 1,927 ng/mL | 58.60 ng/mL | 3.74% |
|  | EGF | WIHS | 0.51 - 62.57 ng/mL | 15.75 ng/mL | 5.98% |
|  |  | MACS | 0.45 - 65.08 ng/mL | 11.48 ng/mL | 2.74% |
|  | NGAL | WIHS | 1.90 - 6,072.84 ng/mL | 89.51 ng/mL | 4.37% |
|  |  | MACS | 0.85 - 294 ng/mL | 20.96 ng/mL | 4.05% |
|  | OPN | WIHS | 16.88 - 7,130.57 ng/mL | 1,191.66 ng/mL | 10.46% |
|  |  | MACS | 24.41 - 7016 ng/mL | 995 ng/mL | 5.94% |
|  | UMOD | WIHS | 646.15 - 27,876 ng/mL | 4,109 ng/mL | 2.69% |
|  |  | MACS | 561 - 64,524 ng/mL | 7870 ng/mL | 2.81% |
| MSD Kidney Injury Panel 3 custom 2-plex (Meso Scale Diagnostics, Gaithersburg, MD) | Clusterin | WIHS | Undetectable - 1,937,752 pg/mL | 225,950 pg/mL | 8.98% |
|  |  | MACS | 200.00 - 2,086,290 pg/mL | 139,900 pg/mL |  |
|  | TFF3 | WIHS | Undetectable - 18,458 pg/mL | 532.00 pg/mL | 10.45% |
|  |  | MACS | Undetectable - 8,373 pg/mL | 462.00 pg/mL |  |
| MSD Kidney Injury Panel 4 custom 4-plex (Meso Scale Diagnostics, Gaithersburg, MD) | IL-18 | WIHS | 2.90 - 1091 pg/mL | 105 pg/mL | 6.19% |
|  |  | MACS | 1.64 - 702 pg/mL | 63 pg/mL |  |
|  | KIM-1 | WIHS | 7.97 - 8216 pg/mL | 1236 pg/mL | 11.68% |
|  |  | MACS | 0.852 - 9897 pg/mL | 988 pg/mL |  |
|  | MCP-1 | WIHS | 2.84 - 2935 pg/mL | 430 pg/mL | 6.53% |
|  |  | MACS | 0.309 - 3033 pg/mL | 286 pg/mL |  |
|  | YKL-40 | WIHS | 11.5 - 381,911 pg/mL | 3938 pg/mL | 4.95% |
|  |  | MACS | 7.63 - 4917 pg/mL | 791 pg/mL |  |
| Siemens BNII nephelometer (Siemens, Munich, Germany) | α1m | WIHS and MACS | 5 - 80 mg/L | n/a | 4.38%, 9.15%, 9.96% |
| Roche Cobas c311 (Roche Diagnostics, Indianapolis, IN) | Creatinine | WIHS and MACS | 1.1 – 610 mg/dL | n/a | 2.25%, 2.29% |

α1m, alpha-1-microglobulin; β2m, beta-2-microglobulin; CysC, cystatin C; CV, coefficient of variation; EGF, epidermal growth factor; IL-18, interleukin-18; KIM-1, kidney injury molecule-1; MCP-1, monocyte chemoattractant protein-1; NGAL, neutrophil gelatinase–associated lipocalin; OPN, osteopontin; UMOD, uromodulin; TFF3, trefoil factor 3; YKL-40, chitinase 3-like protein 1
